# Biodegradation of di-*n*-Butyl Phthalate by *Achromobacter* sp. Isolated from Rural Domestic Wastewater

**DOI:** 10.3390/ijerph121013510

**Published:** 2015-10-26

**Authors:** Decai Jin, Xiao Kong, Yujie Li, Zhihui Bai, Guoqiang Zhuang, Xuliang Zhuang, Ye Deng

**Affiliations:** 1 Key Laboratory of Environmental Biotechnology, Research Centre for Eco-Environmental Sciences, Chinese Academy of Sciences, Beijing 100085, China; E-Mails: dcjin@rcees.ac.cn (D.J.); kongxiaozaikeda@163.com (X.K.); gqzhuang@rcees.ac.cn (G.Z.); xlzhuang@rcees.ac.cn (X.Z.); 2 Environmental Protection Bureau of Shijiazhuang City, Shijiazhuang 050021, China; E-Mail: lyj8026@163.com

**Keywords:** biodegradation, di-*n*-butyl phthalate, environmental hormone, *Achromobacter*, heavy metals, surfactants

## Abstract

A bacterial strain W-1, isolated from rural domestic wastewater, can utilize the environmental hormone di-*n*-butyl phthalate (DBP) as the sole carbon and energy source. The isolated bacterium species was confirmed to belong to the genus *Achromobacter* based on its 16S rRNA gene sequence. The results of substrate utilization tests showed that the strain W-1 could utilize other common phthalates and phenol. High-performance liquid chromatography analysis revealed that the optimal conditions for DBP degradation were pH 7.0, 35 °C, and an agitation rate of 175 rpm. Under these conditions, 500 mg/L of DBP was completely degraded within 30 h. The effects of heavy metals (50 mg/L Cu^2+^ and 500 mg/L Pb^2+^) and surfactants (100 mg/L SDS and 500 mg/L Tween 20) on DBP degradation were investigated. The results demonstrated that Cu^2+^ and SDS severely inhibited DBP degradation and Pb^2+^ weakly inhibited DBP degradation, while Tween 20 greatly enhanced DBP degradation. Furthermore, phthalate degradation genes were found to be located on a plasmid present in *Achromobacter* sp. W-1.

## 1. Introduction

Phthalic acid esters (PAEs) are among the most widely used synthetic chemicals and are employed as additives to improve the flexibility, transparency, durability, and longevity of plastics. PAEs have been detected in soils, water, and air and have become a major environmental concern. Currently, PAEs are listed as primary controlled pollutants by the Environmental Monitoring Center of China and the United States Environmental Protection Agency because of their carcinogenicity and mutagenicity and their ability to act as environmental hormones [1]. As one of the most extensively used PAEs, di-*n*-butyl phthalate (DBP) has become a ubiquitous contaminant in the environment [2]. For example, DBP has been detected in the water and sediment samples with concentration ranges of 1.0–13.5 μg/L and 0.3–30.3 μg/g, respectively [3]. Wang *et al.* [4] reported a total of six priority control phthalates ranging from 0.51 to 7.16 mg/kg in vegetables and 0.40 to 6.20 mg/kg in soils, with average concentrations of 2.56 and 2.23 mg/kg, respectively. DBP, di-(2-ethylhexyl) phthalate (DEHP), and di-*n*-octyl phthalate (DOP) contributed more than 90% to these levels. DBP in the environment can be taken up by plants and other living organisms, thereby entering the food supply. Numerous studies have shown that DBP has anti-androgenic and estrogenic effects in male rats and fish [5], and has also been related to reproductive defects in humans [6]. Since the hydrolysis and photolysis of phthalate esters are very slow, microbial degradation is the main approach used to completely mineralize phthalate esters in the natural environment [7]. Thus, it is critical to isolate microbial strains that efficiently biodegrade DBP in various environments.

Over the past few decades, several phthalate esters-degrading bacterial strains exhibiting diverse geographical distributions have been isolated, including *Rhodococcus* sp. [8], *Agrobacterium* sp. [9], *Gordonia* sp. [10], *Enterobacter* sp. [11], *Pseudomonas* sp. [12], *Burkholderia* sp. [13], *Bacillus* sp. [14], and *Deinococcus radiodurans* and *Pseudomonas stutzeri* [15]. Moreover, considerable research efforts have been devoted to determining the metabolic pathways and mechanisms of PAE biodegradation. In general, the process of PAE biodegradation is as follows: initially, bacteria hydrolyze PAEs into phthalate and the corresponding alcohols, and then phthalate is metabolized to protocatechuate by either phthalate 4, 5-dioxygenase in Gram-negative bacteria or phthalate 3, 4-dioxygenase in Gram-positive bacteria. Finally, protocatechuate is further metabolized into CO_2_ and H_2_O through a series of reactions [16,17]. To date, several genes encoding phthalate-degrading enzymes (esterase, phthalate hydrolase, and phthalate catabolic gene cluster, *etc.*) have been cloned and characterized [17,18,19,20,21]. However, very little information is available regarding the mechanism by which bacterial strains degrade PAEs in rural domestic wastewater. A deeper understanding of this topic could provide a basis for enhancing PAE removal in wastewater treatment plants in rural areas.

In this study, we isolated a bacterial strain W-1 from rural domestic wastewater, which was further identified as a species of the genus *Achromobacter*. We examined the effects of environmental factors on the DBP degradation ability of W-1, analyzed how heavy metals and surfactants affect DBP biodegradation, and investigated the degradation kinetics of DBP at distinct substrate concentrations. Furthermore, we conducted plasmid-elimination experiments in order to determine whether the DBP-degradation genes were located in the plasmid present in W-1.

## 2. Experimental Section

### 2.1. Chemicals

DBP, dimethyl phthalate (DMP), diethyl phthalate (DEP), DOP, DEHP, and phenol at greater than 98% purity were purchased from Alfa Aesar (Ward Hill, MA, USA). Methanol, ethyl acetate, and hexane (high-performance liquid chromatography grade) were purchased from Fisher Scientific (Waltham, MA, USA). All other chemicals and solvents were of analytical reagent grade.

### 2.2. Isolation and Identification of Bacteria

The bacterium was isolated from wastewater obtained from a rural sewage treatment plant in the Huairou District of Beijing (40.3N′, 116.6E′). One milliliter of wastewater was added to 100 mL of mineral salt medium (MSM, g/L: 5.8 K_2_HPO_4_, 4.5 KH_2_PO_4_, 2.0 (NH_4_)_2_SO_4_, 0.16 MgCl_2_, 0.02 CaCl_2_, 0.0024 Na_2_MoO_4_·2H_2_O, 0.0018 FeCl_3_, and 0.0015 MnCl_2_·2H_2_O). The pH of the medium was adjusted to 7.0, and then the medium containing 100 mg/L of PAEs (25 mg/L each of DMP, DEP, DBP, and DEHP) was autoclaved at 115 °C for 30 min,. The culture suspension was incubated for 1–2 weeks at 30°C on a rotary shaker at 175 rpm. Next, 1 mL of the enriched culture solution was transferred into fresh medium containing a higher concentration of PAEs. The final enriched solution was streaked onto MSM agar (18 g/L) plates supplemented with a mixture of PAEs (500 mg/L). Visible colonies appeared after incubation for 1 week. The colonies were then transferred to fresh plates and the incubation process was repeated until pure cultures were obtained. The pure cultures were phylogenetically analyzed by sequencing their 16S rRNA genes, which were PCR-amplified using the universal primers 27F and 1492R. The PCR thermal cycling consisted of an initial denaturation at 95 °C for 10 min, followed by 35 cycles of 94 °C for 45 s, 55 °C for 45 s, and 72 °C for 90 s, plus a final step at 72 °C for 10 min. PCR products were cloned and sequenced. The obtained sequences were subjected to BLAST homology searching (http://www.ncbi.nlm.nih.gov/BLAST). Based on the sequencing results, the 16S rRNA gene sequences of higher similarity-related strains from GenBank database were selected. Multiple sequence alignment was performed and a phylogenetic tree was generated using MEGA4.0 software.

### 2.3. Substrate Utilization Tests

To examine the ability of strain W-1 to utilize different substrates, W-1 was cultured in liquid MSM supplemented with 500 mg/L of one of the following substrates: DMP, DEP, DBP, DOP, DEHP, and phenol as the sole source of carbon and energy. The suspension was incubated at 30 °C on a rotary shaker at 175 rpm. After 3 days of incubation, the biomass concentration was determined by optical density measurements at 600 nm.

### 2.4. Effects of pH, Temperature, and Agitation Rate on DBP Biodegradation

Strain W-1 was cultivated in MSM at 30°C on a rotary shaker at 175 rpm, harvested after 48 h, washed three times with 0.02 M potassium phosphate buffer (pH 7.2) under sterile conditions, and resuspended in the same phosphate buffer to an OD_600_ of 0.2. A stock solution of 10 g/L DBP was prepared by dissolving DBP in *n*-hexane, and then equal amounts of the solutions were added to 50 mL sterilized Erlenmeyer flasks. The *n*-hexane was evaporated and then the samples were inoculated with 1 mL of the cell suspension prepared in 20 mL of MSM. To determine the optimal conditions for biodegradation, the degradation of 500 mg/L DBP by W-1 was examined at different pH (5.0, 6.0, 7.0, 8.0, 9.0, 10.0, and 11.0), temperatures (25, 30, 35, 40, and 45 °C), and agitation rates (0, 75, 150, 175, and 200 rpm). After incubation for 30 h, samples were collected and subjected to high-performance liquid chromatography (HPLC) analysis. All experiments were performed in triplicate.

### 2.5. Effects of Heavy Metals and Surfactant on DBP Degradation by Strain W-1

Because heavy metals and surfactants are present in soil and water, they may influence the degradation of organic matter by microorganisms. Therefore, we investigated the effects of heavy metals (Cu^2+^ and Pb^2+^) and surfactants (SDS and Tween 20) on the degradation of DBP by W-1; the following concentrations were tested: 50 mg/L Cu; 500 mg/L Pb; 100 mg/L SDS; and 500 mg/L Tween 20. The experiment was conducted in 50-mL Erlenmeyer flasks containing 20 mL of MSM supplemented with 500 mg/L of DBP. The culture was incubated on a rotary shaker at 175 rpm and 30 °C, and samples were withdrawn once every 8 h until 48 h. The DBP residue was detected using HPLC. All experiments were performed in triplicate.

### 2.6. Kinetics of DBP Degradation by Strain W-1

The degradation of DBP at various initial substrate concentrations (100, 200, 300, 400, and 500 mg/L) was examined under optimal conditions. Samples were collected once every 5 h for a period of 30 h, and HPLC analysis was performed to detect the DBP residue. All experiments were performed in triplicate.

### 2.7. Plasmid Eliminate Experiment

Plasmids were eliminated as described previously [22]. Briefly, the bacterial strain was inoculated in 20 mL of LB medium without or with 0.01% SDS. Cells were cultivated at 35 °C and 175 rpm for 16 h, and the cultivation was repeated 3 times under this condition. The diluted bacteria were coated on LB agar medium and grown at 35 °C for 24 h. Single colonies were propagated and the plasmids were extracted; plasmid isolation was repeated twice. Plasmid-removed cells and normal cells were inoculated into 100 mL of MSM containing 500 mg/L DBP and grown at 35 °C and 175 rpm, and the growth of these bacterial cells and their DBP degradation were compared.

### 2.8. Analysis of DBP Residue

A total of 20 mL of ethyl acetate was added directly to the flask, which was shaken up and then left standing until stratification. The ethyl acetate phase without bacteria was evaporated to dryness and dissolved in 10 mL of methanol. The methanol solution was filtered through a 0.22-µm membrane filter before injection into the HPLC system equipped with a UV230+ UV-Vis detector and a Hypersil BDS-C18 (200 mm × 4.6 mm, 5 µm) chromatography column. The chromatographic conditions for detecting DBP were mobile phase: 9:1 (v/v) methanol-water, flow rate of 1 mL/min, and UV wavelength of 228 nm.

## 3. Results and Discussion

### 3.1. Isolation and Identification of Bacteria

**Figure 1:**
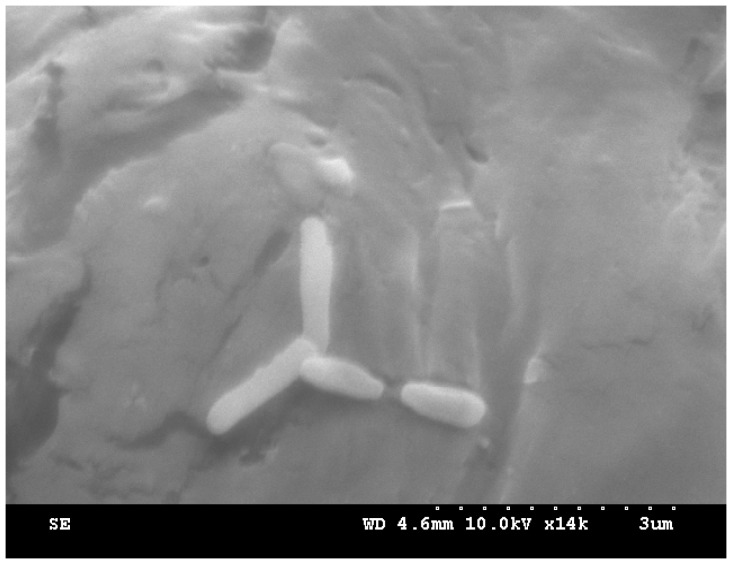
Scanning electron micrograph of strain W-1.

A bacterial strain (designated as W-1) that can use DBP was enriched and isolated from rural domestic wastewater obtained from a wastewater-treatment plant. The properties of the original water were as follows: pH = 7.60, temperature = 12.2 °C, salinity = 566 mg/L, COD = 346 mg/L, SS = 26 mg/L, anionic surfactant = 2.00 mg/L, TN = 54.6 mg/L, TP = 4.97 mg/L, Cl^−^ = 44.2 mg/L, and total bacterial count = (1.24 ± 0.05)×10^6^/mL. Strain W-1 was found to be a Gram-negative bacterium and its colonies were round, moist, neat, and smooth, ranging in color from white to light brown when grown on LB agar. The bacterium could utilize glucose, mannose, citrate, sorbitol, asparagine, and proline as sole carbon sources. Maltose, soluble starch, malonic acid, tryptophan, cysteine, and serine were not utilized. As shown in Figure 1, the size of strain W-1 was approximately 0.3–0.6 μm × 1.2–1.8 μm.

**Figure 2:**
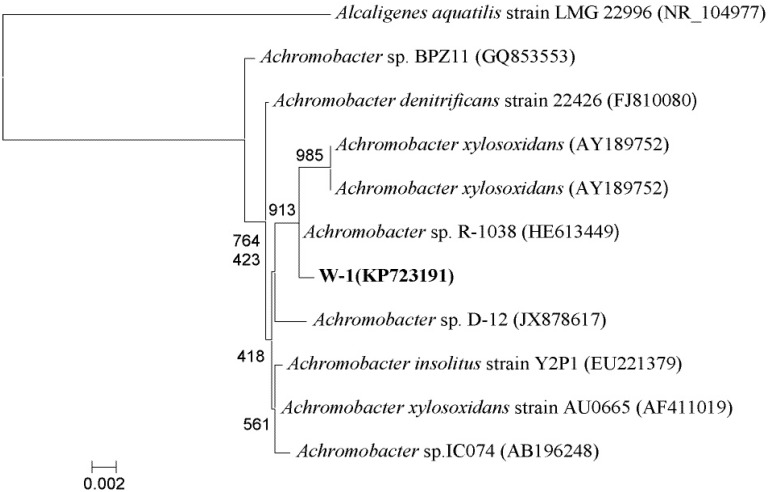
Phylogenetic tree derived from the 16S rRNA gene sequence of strain W-1 and sequences of related species. Distances were calculated using the neighbor-joining method. Numbers at branch points are bootstrap values (based on 1000 samplings). *Alcaligenes aquatilis* strain LMG 22996T (NR_104977) was used as the out-group. Scale bars represent 0.002 substitutions per site.

A BLAST analysis of the 16S rRNA gene sequence of W-1 (GenBank accession no. KP723191) revealed that this strain and several *Achromobacter* strains share an extremely high degree of homology (>99%). Thus, strain W-1 was identified as belonging to the genus *Achromobacter*. The relationship between *Achromobacter* sp. strain W-1 and its close relatives based on 16S rRNA gene sequence analysis is shown in Figure 2. Although previous studies have shown that *Achromobacter* species can degrade *p*-nitrophenol [23], 2,4-dichlorophenol and phenol [24], acetochlor [25], and reduce high concentrations of Cr [26], only one recent study reported PAE degradation by pure cultures of *Achromobacter* [27].

### 3.2. Substrate Utilization Tests

Domestic wastewater typically contains diverse pollutants, and thus screening for strains that can degrade a broad spectrum of substrates is highly conducive for using these strains in practical engineering. The results of substrate utilization tests indicated that the isolate W-1 degraded various phthalate esters (Table 1). The strain grew well in media containing DMP, DEP, DBP, DOP, and DEHP, demonstrating that W-1can utilize both short alkyl-chained PAEs and long alkyl-chained PAEs. Notably, strain W-1 can grow better in long alkyl-chained than in short alkyl-chained PAEs. Moreover, W-1 also used phenol, which is a widespread pollutant worldwide. Based on these results, DBP, which is one of most popular plasticizers, was used in further degradation tests [28].

**Table 1:** Substrate utilization profile of strain W-1.

Substrate	DMP/OD_600_	DEP/OD_600_	DBP/OD_600_	DEHP/OD_600_	DOP/OD_600_	Phenol/OD_600_
Utilization	+/0.340	+/0.426	+/0.537	+/1.179	+/0.962	+/0.902

Note: +, positive.

### 3.3. Effects of pH, Temperature, and Agitation rate on DBP Biodegradation

**Figure 3:**
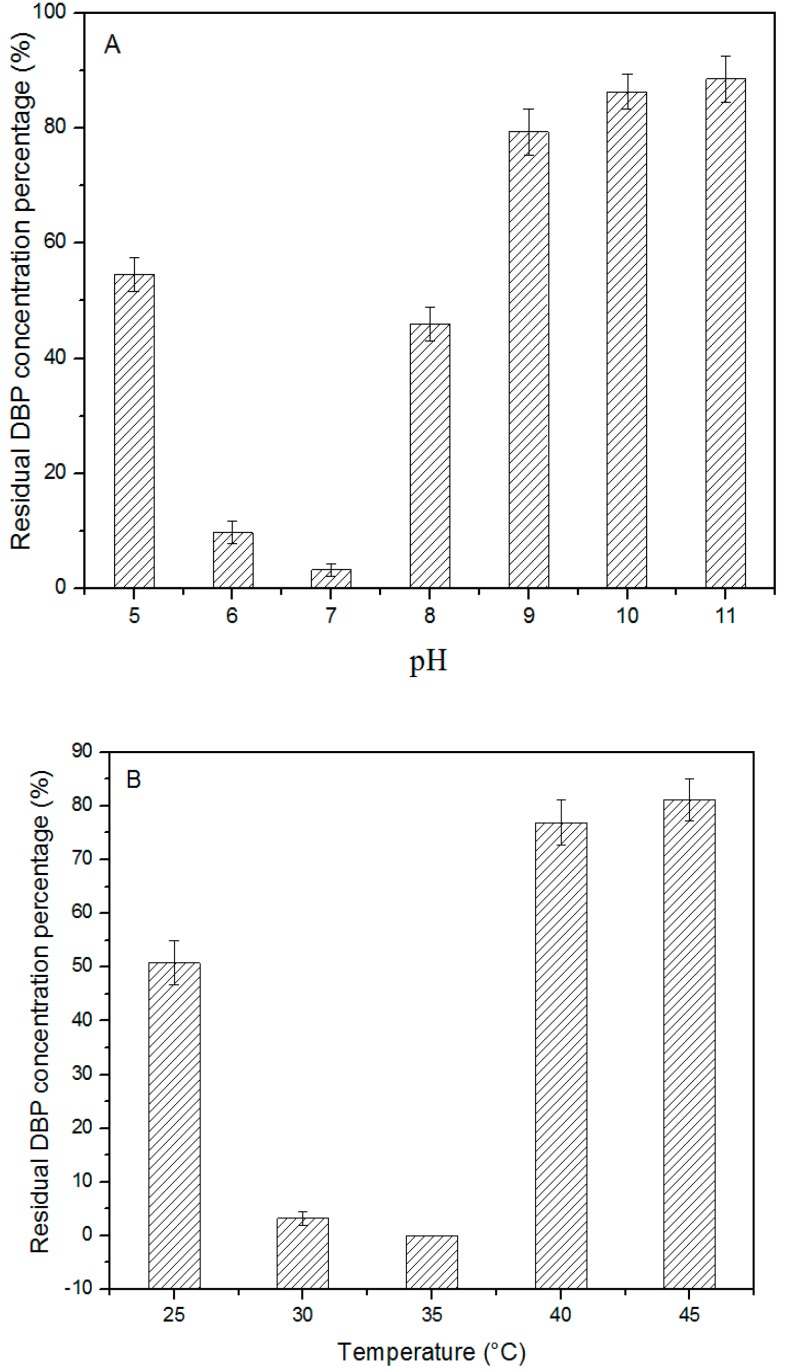

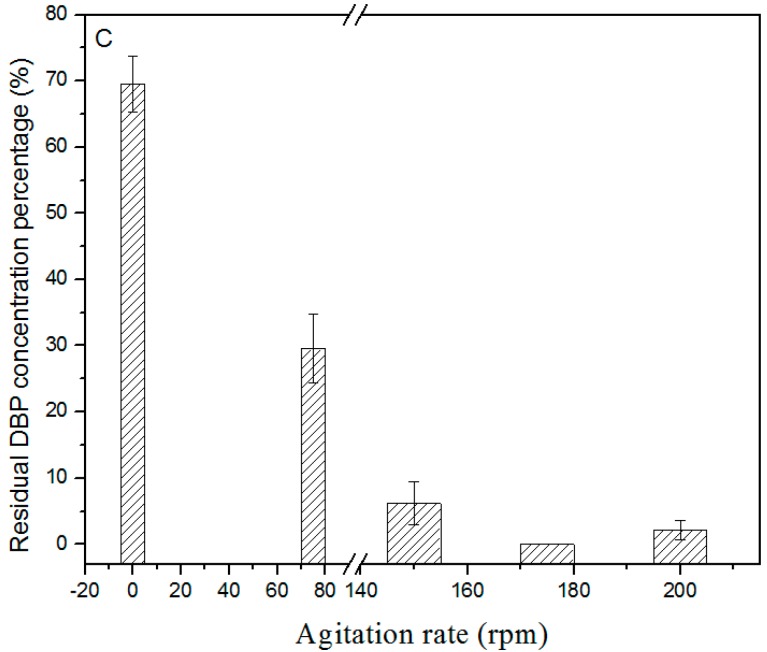
Effect of pH, temperature, and agitation rate on degradation of DBP (500 mg/L) by strain W-1. A: pH; B: temperature; C: agitation rate; Error bars are the standard error of the mean (SEM) of DBP concentration from triplicate experiments.

The effects of pH on DBP degradation by strain W-1 in the batch medium were tested at 30 °C, and the results are shown in Figure 3. The DBP degradation rate increased rapidly when the pH was increased from 5.0 to 7.0. The highest DBP degradation rate (approximately 96.8%) for strainW-1 was achieved at pH 7.0. However, the degradation rate decreased dramatically when pH exceeded 7.0. In the temperature effect test, the degradation rate increased when the temperature was increased from 25 to 35 °C, but the rate decreased at higher temperatures. Therefore, the optimal temperature for DBP degradation was 35 °C. Finally, the DBP-degradation rate decreased when the agitation rate was increased from 0 to 175 rpm, but it decreased when the agitation rate was >175 rpm. Collectively, these results revealed the optimal conditions for DBP degradation as pH 7.0, 35 °C, and agitation rate of 175 rpm. No residual DBP was detected under this set of conditions within 30 h. Pradeep *et al.* [27] reported that the optimal conditions for DEHP degradation by *Achromobacter denitrificans* strain SP1 were 32 °C, agitation rate of 200 rpm, and pH 8.0; thus, strain W-1 and *A. denitrificans* strain SP1 exhibited similar trends with respect to optimal conditions.

### 3.4. Effects of Heavy Metals and Surfactants on DBP Degradation by Strain W-1

**Figure 4:**
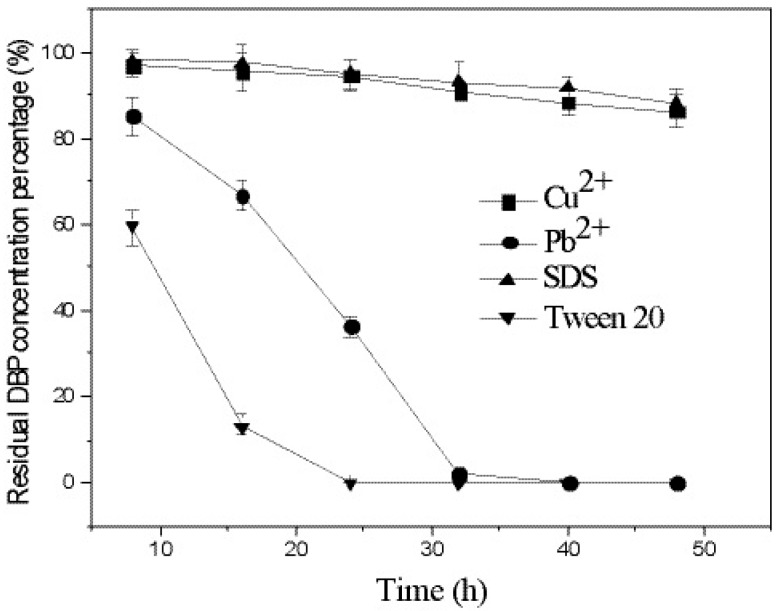
Effects of heavy metals and surfactants on the degradation of DBP. Error bars are the SEM of DBP concentration from triplicate experiments.

We examined how the degradation of DBP in culture medium was affected by the heavy metals Cu^2+^ (50 mg/L) and Pb^2+^ (500 mg/L), and the surfactants SDS (100 mg/L) and Tween 20 (500 mg/L). The results presented in Figure 4 show that strain W-1 completely degraded DBP in the absence of Cu^2+^ or Pb^2+^ within 30 h. However, W-1 was extremely sensitive to Cu^2+^ and SDS: almost no DBP degradation occurred in their presence. When Pb^2+^ was added to the reaction mixture, no marked effects on DBP degradation were observed; 98.2% of the DBP was degraded by 32 h and no DBP was detected at 40 h. Finally, Tween 20 substantially enhanced DBP degradation: no DBP was detected by 24 h when this surfactant was present. These results indicate that various environmental factors must be considered when conducting bioremediation of sites contaminated with PAEs.

### 3.5. Kinetics of DBP Degradation by Strain W-1

**Figure 5:**
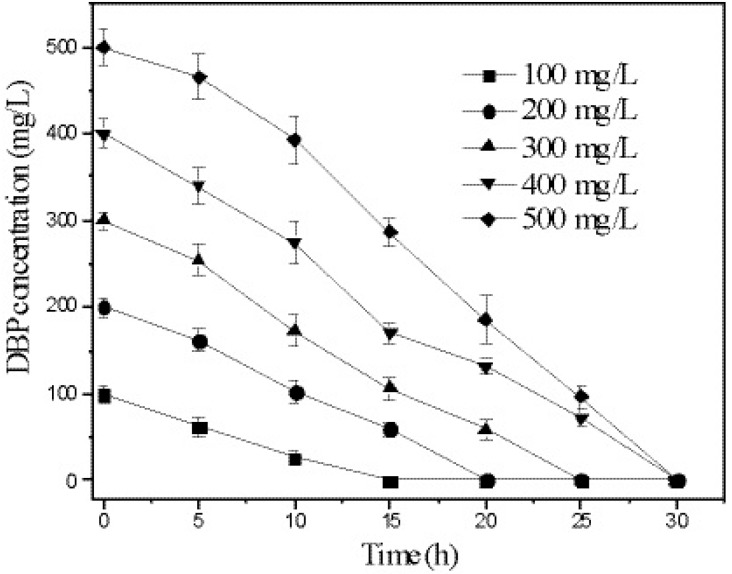
Degradation rate at different initial DBP concentrations. Error bars are the SEM of DBP concentration from triplicate experiments.

The degradation of DBP by strain W-1 at different initial concentrations was investigated. The results (Figure 5) show that strain W-1 completely degraded DBP within 20 h when the initial concentration was less than 200 mg/L. When the initial concentration of DBP was between 300 and 500 mg/L, all DBP was degraded within 30 h.

DBP biodegradation by strain W-1 was assumed to fit the following first-order kinetic model: ln *C* = −*k*t + b (1), where *C* is DBP concentration, t is time, *k* is the first-order rate constant, and b is a constant. The half-life of DBP biodegradation by strain W-1 was calculated according to the following equation: *t*_1/2_ = $\ln\frac{2}{k}$ (2). The results in Table 2 show that the DBP-depletion curves were described accurately by the first-order kinetic equation, with high correlation coefficients (R^2^ > 0.96) at initial DBP concentrations ranging from 100 to 400 mg/L. To date, the first-order kinetic model has been used most widely to describe the biodegradation of PAEs by bacterial strains. Xu *et al.* [12] reported that DBP biodegradation by *Pseudomonas fluorescens* B-1 at very low DBP concentrations (2.5–10.0 mg/L) could be accurately described using the first-order kinetic model, and Wu *et al.* [9] reported that DBP degradation by *Agrobacterium* sp. fit well with first-order kinetics and that the half-life of degradation was 27.05 h when the DBP concentration was 500 mg/L. Therefore, DBP degradation by strain W-1 was more efficient than reported previously for other microbial strains.

**Table 2:** Kinetics of DBP degradation by strain W-1.

Initial Concentration (mg/L)	Kinetic Equations	R^2^	t_1/2_ (h)
100	ln*C* = −0.1341 x + 4.6721	0.9709	5.1
200	ln*C* = −0.0826 x + 5.3931	0.9642	8.3
300	ln*C* = −0.0825 x + 5.8534	0.9623	8.4
400	ln*C* = −0.0674 x + 6.1341	0.9610	10.3
500	ln*C* = −0.0644 x + 6.4367	0.9109	10.8

### 3.6. Plasmid Elimination Experiment

**Figure 6:**
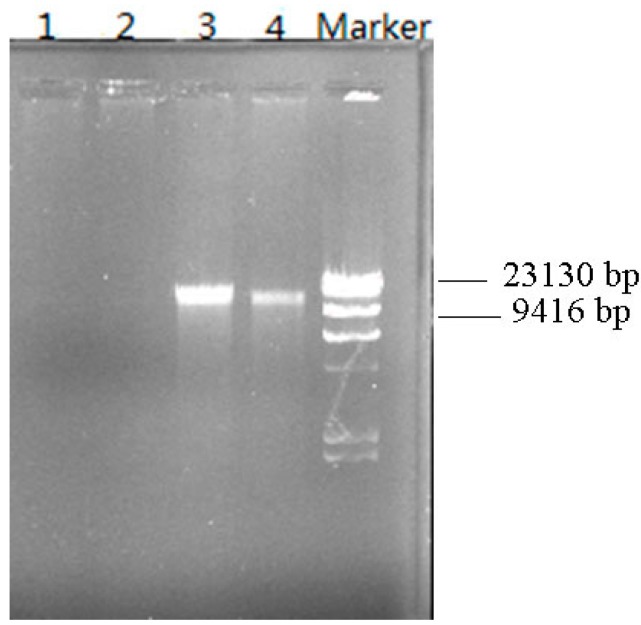
Plasmid DNA electrophoresis before and after the plasmid-elimination test. 1–2: Plasmid DNA after plasmid elimination; 3–4: Plasmid DNA before elimination.

It is well-known that plasmids plays an important role in the degradation of pollutants and can be useful for genetic bioaugmentation. The plasmid DNA in strain W-1 was eliminated using a series of steps. Figure 6 shows the plasmid DNA electrophoresis before and after the plasmid elimination test. In strain W-1, one plasmid DNA with a size <23 kb was identified. The results of the degradation experiment showed that W-1 could not degrade DBP and other PAEs (DMP, DEP, DOP, and DEHP) after plasmid elimination, strongly indicating that the degradation of DBP is mediated by plasmid-encoded enzymes in strain W-1. Plasmids occur naturally in several species of the genus *Achromobacter*. For instance, in 2000, a 70-kb plasmid, pEST4011, was shown to be responsible for the degradation of 2,4-dichlorophenoxyacetic acid by *A. xylosoxidans* subsp. *denitrificans* strain EST4002 [29]. In 2011, a 7-Mb chromosome and two large plasmids (98 and 248 kb) were identified in the genome of *A. xylosoxidans* strain A8, which can use 2-chlorobenzoate and 2,5-dichlorobenzoate as the sole sources of carbon and energy [30]. Moreover, plasmids are widely known to play critical roles in the bacterial degradation of synthetic compounds. With respect to PAEs, Nomura *et al.* [31] reported a 7-kbp plasmid in *Pseudomonas putida* encoding the enzymes responsible for the initial phthalate degradation in 1992; later, a large plasmid of approximately 140 kb involved in the degradation of phthalates was found in *Pseudomonas fuorescens* [32]. In 2001, Eaton [18] reported a plasmid-encoded phthalate catabolic pathway in *Arthrobacter keyseri* 12B. However, there is little information available regarding PAE degradation mediated by plasmid-encoded enzymes. Thus, our results may enhance the understanding of PAE-degradation mechanisms and the functional diversity in the genus *Achromobacter*.

## 4. Conclusions

The bacterial strain W-1, which was capable of using DBP as the sole carbon and energy source, was isolated from rural wastewater and identified as *Achromobacter* sp. based on its 16S rRNA gene sequences. This is the first report of using an *Achromobacter* sp. for DBP degradation. The optimal DBP degradation conditions were pH 7.0, 35 °C, and agitation rate of 175 rpm. The kinetics of DBP degradation at various initial DBP concentrations (100–400 mg/L) could be described accurately using a first-order kinetic model. DBP degradation was strongly inhibited by Cu^2+^ and SDS and weakly inhibited by Pb^2+^, but the degradation was potently enhanced by Tween 20. The degradation of DBP and other PAEs was controlled by a single plasmid present in strain W-1. These results suggest that *Achromobacter* sp. W-1 can be used as a potential candidate for the efficient bioremediation of PAE-contaminated soils and water. Based on our findings, future studies should examine the mechanism of PAE degradation mediated by plasmid-encoded enzymes.
